# Topic Discovery and Hotspot Analysis of Sentiment Analysis of Chinese Text Using Information-Theoretic Method

**DOI:** 10.3390/e25060935

**Published:** 2023-06-13

**Authors:** Changlu Zhang, Haojie Fan, Jian Zhang, Qiong Yang, Liqian Tang

**Affiliations:** 1 School of Economics & Management, Beijing Information Science & Technology University, Beijing 100192, China; 20151935@bistu.edu.cn (C.Z.); 2021020730@bistu.edu.cn (Q.Y.); 2022020740@bistu.edu.cn (L.T.); 2 Beijing Key Lab of Green Development Decision Based on Big Data, Beijing 100192, China; 2021020681@bistu.edu.cn; 3 School of Computer Science, Beijing Information Science & Technology University, Beijing 100192, China

**Keywords:** sentiment analysis, topic discovery, FastText, information gain, hierarchical clustering

## Abstract

Currently, sentiment analysis is a research hotspot in many fields such as computer science and statistical science. Topic discovery of the literature in the field of text sentiment analysis aims to provide scholars with a quick and effective understanding of its research trends. In this paper, we propose a new model for the topic discovery analysis of literature. Firstly, the FastText model is applied to calculate the word vector of literature keywords, based on which cosine similarity is applied to calculate keyword similarity, to carry out the merging of synonymous keywords. Secondly, the hierarchical clustering method based on the Jaccard coefficient is used to cluster the domain literature and count the literature volume of each topic. Thirdly, the information gain method is applied to extract the high information gain characteristic words of various topics, based on which the connotation of each topic is condensed. Finally, by conducting a time series analysis of the literature, a four-quadrant matrix of topic distribution is constructed to compare the research trends of each topic within different stages. The 1186 articles in the field of text sentiment analysis from 2012 to 2022 can be divided into 12 categories. By comparing and analyzing the topic distribution matrices of the two phases of 2012 to 2016 and 2017 to 2022, it is found that the various categories of topics have obvious research development changes in different phases. The results show that: ① Among the 12 categories, online opinion analysis of social media comments represented by microblogs is one of the current hot topics. ② The integration and application of methods such as sentiment lexicon, traditional machine learning and deep learning should be enhanced. ③ Semantic disambiguation of aspect-level sentiment analysis is one of the current difficult problems this field faces. ④ Research on multimodal sentiment analysis and cross-modal sentiment analysis should be promoted.

## 1. Introduction

Text sentiment analysis is the process of analyzing and mining textual data to obtain information about people’s opinions, views, attitudes and sentiments [1]. Research in this area began with Pang et al.’s supervised learning approach to classify the sentiment of movie review texts [2]. Along with the rapid development of mobile Internet and the widespread use of mobile terminals, text sentiment analysis has gradually become one of the most active research areas in natural language processing, text mining and information retrieval, and has attracted the attention of all of society due to its important academic value and commercial application value.

In recent years, research results on Chinese text sentiment analysis have been emerging in domestic academia. Especially since the release of Word2Vec by Google in 2013, research related to sentiment analysis has entered a rapid development stage, with more than 700 relevant articles retrieved on the China National Knowledge Infrastructure (CNKI) and more than 2500 relevant articles in Web of Science in 2022 alone. Some representative achievements are as follows. Alslaity et al. proposed a systematic review of 123 papers on machine-learning-based emotion detection to investigate research trends along multiple themes, including machine learning approaches, application domains, data, evaluation, and outcomes [3]. Bonifazi et al. presented a general framework for extracting information about the scope of the sentiment of a user on topics of any subject; this framework is capable of operating on any social platform [4]. In their study, Almars et al. proposed a novel probabilistic model, called the hierarchical user sentiment topic model (HUSTM), to discover the hidden structure of topics and users while performing sentiment analysis in a unified way [5]. Yin et al. proposed a study aimed at exploring Chinese people’s attitudes towards volunteerism in response to the urgent need for volunteers to complement overwhelmed public health systems during the COVID-19 pandemic. To achieve this goal, they conducted a topic modeling analysis using latent Dirichlet allocation (LDA) on volunteerism-related microblogs posted on Weibo, the Chinese equivalent of Twitter [6]. Jelodar et al. proposed a novel content analysis to examine user reviews or movie comments on YouTube. In fact, the proposed hybrid framework is based on semantic and sentiment aspects using fuzzy lattice reasoning to meaningful latent-topic detection and utilizing the sentiment analysis of user comments of Oscar-nominated movie trailers on YouTube. Based on the word vector feature, classification algorithms are employed to detect the comments’ sentiment level. The results of this study suggest that this hybrid framework could be effective in extracting associated features and latent topics with sentiment valence on user comments [7].

The use of scientific literature analysis methods to discover and analyze the emerging literature in this field is conducive to revealing the current topic distribution, hotspot areas and research shortcomings, and looking into directions for future development, which are of great significance in guiding the future development of related theories and technologies, and the expansion of application scenarios. Current research that systematically composes the literature in the field of text sentiment analysis can be divided into two categories.

The first type is the reviewed literature, which is a description of a literature review in the field of text sentiment analysis from a qualitative analysis perspective, including concepts, current research status, research methods, existing problems, and future trends. Representative research achievements, such as those of Hong et al., elaborated on sentiment lexicon methods, supervised machine learning methods, weakly supervised deep learning methods, and other methods from the perspective of text sentiment analysis [8]. Wang et al. explained the technical methods of image sentiment analysis and pointed out that the future research direction lies in optimizing deep learning algorithms and annotation methods [9]. Liu et al. reviewed the multimodal sentiment analysis technology and summarized the challenges and development trends faced by multimodal sentiment analysis in the future [10]. Xu et al. sorted out the research status of the Russian sentiment analysis field and compared and analyzed the performance and feature selection schemes of various methods on different datasets [11]. Tan explained the impact and promotion of fine-grained sentiment analysis on the entire sentiment analysis method and summarized the latest tasks and technical methods of fine-grained text sentiment analysis [12].

The second type is literature related to measurement. This type of literature mainly uses bibliometric analysis as its main method, and discusses the existing sentiment analysis literature from the perspectives of literature time series, major research institutions, discipline distribution, and hot topic mining through statistical analysis. Representative research results include the following: Zhao et al. systematically sorted out the number of publications, disciplinary classification, and hot topics in the field of sentiment analysis at home and abroad based on bibliometric and visualization methods [13]. Chen et al. statistically analyzed domestic journals and author teams in the field of text sentiment analysis, used co-occurrence analysis to study the relationship between keywords, and explored the research hotspots and current situation of text sentiment analysis [14]. Tan et al. used CiteSpace to construct a visualization knowledge map of sentiment analysis, analyzed the current situation of sentiment analysis in domestic and foreign network public opinion, and compared their differences [15].

Based on the literature review above, it is not difficult to find that previous literature review methods mainly relied on qualitative analysis and summarization, which inevitably led to problems such as subjectivity and insufficient condensation. In recent years, bibliometric methods have been widely used to summarize previous literatures. However, the research methods and perspectives are similar. For example, researchers usually use Citespace to statistically measure the trend, researcher collaboration, and highly cited literature in a certain field. To analyze the research hotspots, researchers typically rely on the frequency and centrality of the keywords. This is actually unreasonable to a certain extent because multiple keywords may belong to the same topic. Therefore, relying solely on the frequency and centrality of keywords to reflect research hotspots has certain drawbacks. In this study, we will firstly construct the topic discovery model with the combination of FastText, hierarchical clustering, and information gain. Then, using the topic discovery model, we try to condense and analyze the topics of text-sentiment-analysis-related literature in order to explore the topic distribution, hot topics, and future trends in the field of sentiment analysis.

## 2. Construction of the Topic Discovery Model Using Information-Theoretic Method

In references [16,17], a topic discovery analysis method based on systematic clustering and information gain was proposed. This method first constructs a literature keywords matrix and then uses the Jaccard coefficient to perform clustering analysis on the matrix. The topics are divided based on the co-occurrence relationships of keywords in different literatures [16,17]. However, this method ignores the problem of synonymy that generally exists in the literature keywords matrix, which ultimately affects the clustering effect.

**Figure 1:**
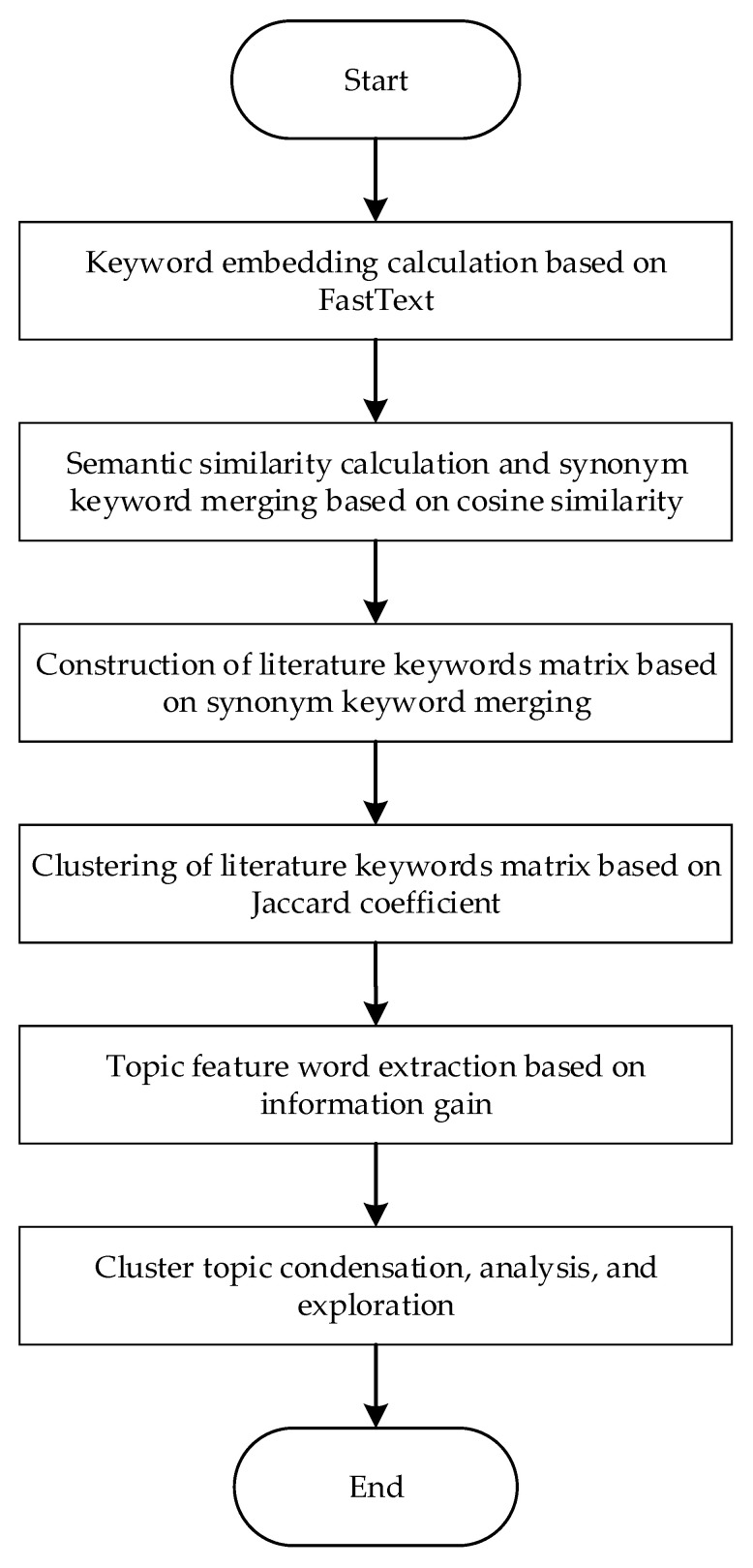
The overall framework of the topic discovery model.

To address this issue, this study proposed a literature topic discovery model based on text mining. Firstly, the FastText model was used to calculate keyword embedding based on the abstract text of the literature as the training corpus. Secondly, the semantic similarity of keyword embedding was calculated using cosine similarity, and synonym keywords were semantically merged to construct a bag of synonym keywords. Thirdly, the literature keywords matrix was generated based on the bag of synonym keywords and then subjected to clustering analysis. Finally, the information gain method was used to calculate the information gain of each keyword for each topic, and the key feature words of each topic were extracted based on the size of the information gain value, followed by refining the topic content. The overall framework of this model is shown in Figure 1.

### 2.1. Synonymous Keyword Merging Based on FastText Model

**Figure 2:**
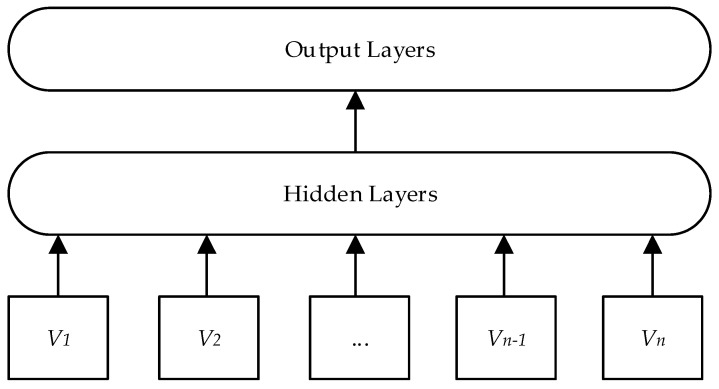
Structure of the FastText model.

FastText is an open-source and efficient word vector calculation and text classification tool, with a three-layer model structure similar to Word2Vec’s Continuous Bag-of-Words (CBOW) model, as shown in Figure 2. Where *V*_1_, *V*_2_, …, *V_n −_*_1_, *V_n_* denotes the word vector proposed by N-gram, and then the average of the summed word vectors is used as the text representation through the hidden layer.

FastText uses layered Softmax and N-gram to extract word sense information, decomposing each word in the input context using an N-gram format, making an effective improvement in the grammatical morphological structure of words. In addition, compared to Word2Vec, which predicts intermediate words through context, the FastText model predicts text labels through all features. After integrating N-gram [18], it has the advantage of providing better word vectors for some rare keywords, effectively solving the problem of individual literature keywords exceeding the training corpus, improving the merging effect of semantically similar keywords, and thereby improving the subsequent clustering effect, making the literature clustering theme more distinct.

Based on the word vectors obtained from the segmented corpus trained by the FastText model, the cosine similarity calculation method was used to calculate the similarity between any two keywords. The calculation is shown in Formula (1):

(1) $$\cos(\theta )=\frac{v_{A}\cdot v_{B}}{\left\|v_{A}\right\|\cdot \left\|v_{B}\right\|}=\frac{\sum_{i=1}^{n}\left(v_{A}\times v_{B}\right)}{\sqrt{\sum_{i=1}^{n}{\left(v_{A_{i}}\right)}^{2}}\times \sqrt{\sum_{i=1}^{n}{\left(v_{B_{i}}\right)}^{2}}}$$

where cos(*θ*) is the cosine value between two keywords, the vector of keyword A is $v_{A}=[v_{A_{1}},\;v_{A_{2}},\;\ldots ,\;v_{A_{i}},\;\ldots ,\;v_{A_{n}}]$, the vector of keyword B is $v_{B}=[v_{B_{1}},\;v_{B_{2}},\;\ldots ,\;v_{B_{i}},\;\ldots ,\;v_{B_{n}}]$, and *n* represents the dimension of the word vector. The value of cos(*θ*) ranges from −1 to 1. The larger the value, the smaller the angle between the two keywords, indicating a smaller difference and higher semantic similarity. When cos(*θ*) is equal to 1, it indicates that the two keywords have an identical relationship. Conversely, the smaller the value of cos(*θ*), the lower the similarity between the two keywords.

### 2.2. Topic Clustering Based on Jaccard Coefficient

The Jaccard coefficient is a statistic used to compare the similarity of sample sets and is a commonly used method for calculating text similarity in the field of computer science. It is defined as the size of the intersection of the sample sets divided by the size of their union [19]. Using the Jaccard coefficient to measure the similarity of literature topics, let *A* and *B* be the sets of keywords contained in two literatures, respectively. The Jaccard coefficient between *A* and *B* is defined as Formula (2):

(2) $$J(A,B)=\frac{\left|A\cap B\right|}{\left|A\cup B\right|}=\frac{\left|A\cap B\right|}{\left|A|+|B|-|A\cap B\right|}$$

Here, *|A|* represents the number of keywords in one literature, *|B|* represents the number of keywords in another literature, *|A∪B|* represents the total number of all the keywords contained in these two literatures, and *|A∩B|* represents the number of keywords that these two literatures have in common.

The larger the Jaccard coefficient, the more keywords the two literatures have in common, and the higher the similarity of the literature topics. Compared to the commonly used simple matching coefficient method, the Jaccard coefficient does not consider the keywords that are not included in both literatures at the same time. This avoids the influence of the sparsity of the literature keywords matrix on the measurement of literature similarity, and has high accuracy and easy-to-understand results, thus achieving an ideal clustering effect.

### 2.3. Topic Feature Word Extraction Based on Information Gain

To accurately analyze the connotation of each research topic based on literature clustering analysis, it is necessary to extract key feature words that reflect the corresponding topic and then condense the topic based on them.

In this study, the information gain method was adopted to measure the contribution of each keyword to the corresponding topic category based on the information brought by each keyword to the topic category. If a keyword brings more information gain, it is more important for the topic and can distinguish the topic from other topics, thus reflecting the connotation of the topic. Generally, the information gain of a keyword *t_k_* for a category *C* is equal to the difference between the information entropy of category *C* without considering the keyword *H(C)* and the information entropy of category *C* when considering the keyword *H(C|t_k_)*.

When the keyword is not taken into account, the information entropy of category *C*, denoted as *H(C)*, is as shown in Formula (3):

(3) $$H(C)=-[p_{c}\log(p_{c})+p_{\bar{c}}\log(p_{\bar{c}})]=-[\frac{n_{1}}{n}\log(\frac{n_{1}}{n})+\frac{n_{2}}{n}\log(\frac{n_{2}}{n})]$$

where *p_c_* is the probability of the occurrence of category *C*, $p_{\bar{c}}$ is the probability of the occurrence of non-category *C*, *n* is the total number of literatures, *n_1_* is the number of literatures belonging to category *C*, and *n_2_* is the number of literatures not belonging to category *C*.

The information entropy *H(C|t_k_)* of category *C* after considering the keyword *t_k_* is shown in Formula (4):

(4) $$\begin{array}{l} H(C\;|\;t_{k})=-\frac{m_{1}+m_{2}}{n}[\frac{m_{1}}{m_{1}+m_{2}}\times \log(\frac{m_{1}}{m_{1}+m_{2}})+\frac{m_{2}}{m_{1}+m_{2}}\times \log(\frac{m_{2}}{m_{1}+m_{2}})]\\ -\frac{m_{3}+m_{4}}{n}[\frac{m_{3}}{m_{3}+m_{4}}\times \log(\frac{m_{3}}{m_{3}+m_{4}})+\frac{m_{4}}{m_{3}+m_{4}}\times \log(\frac{m_{4}}{m_{3}+m_{4}})] \end{array}$$

where *m_1_* is the number of literatures in which keyword *t_k_* appears in category C, *m_2_* is the number of literatures in which keyword *t_k_* appears in non-category *C*, *m_3_* is the number of literatures in which keyword *t_k_* appears in category *C*, and *m_4_* is the number of literatures in which keyword *t_k_* appears in non-category *C*.

Therefore, the information gain of keyword *t_k_* on topic category *C* is shown in Formula (5):

(5) $$IG(t_{k})=H(C)-H(C\;|\;t_{k})$$

## 3. Experiments and Results

### 3.1. Data Sources and Experimental Procedures

A literature search was conducted in the China National Knowledge Infrastructure (CNKI) Journals Database with the subject term “text sentiment analysis”. The search date was 1 January 2023, and the publication time range was set from 2012 to 2022. The source types were Peking University Core and CSSCI, and 1186 articles were finally retrieved. The title, abstract and keywords information of 1186 documents were collected and processed to extract a total of 2272 initial keywords.

The experimental process of this study is described as follows.

Firstly, the abstract text collection in the retrieved literature was divided using Jieba word separation, and the deactivated words were removed from the divided abstract text collection using the Harbin Institute of Technology (HIT) stop words list to form a corpus for keyword embedding training.

Secondly, the FastText model training was conducted to calculate the word vector of each keyword. Additionally, the cosine similarity of any keyword pair was calculated, and the keywords with high similarity were semantically merged to construct the keyword bag of words.

Thirdly, a literature keywords matrix was constructed based on the keyword bag of words, and literature topic clustering was performed based on the Jaccard coefficient.

Fourthly, the information gain of each keyword to each topic category was calculated, and the topic connotation was condensed and analyzed by the high information gain feature words.

Fifthly, the annual distribution of literature on each topic and the four-quadrant matrix of topic distribution were constructed to compare and analyze the research trends.

### 3.2. Experimental Results and Analysis

#### 3.2.1. Keyword Semantic Similarity Calculation and Synonym Merging

The keyword embedding was trained using the FastText model with a vector dimension of 300 dimensions. On this basis, we calculated the cosine similarity of any keyword pair and obtained the keyword semantic similarity matrix, as shown in Table 1.

We merged similar keywords using the semantic similarity matrix of keywords in Table 1. In order to ensure that keywords with high semantic similarity are found during merging, this study set a semantic similarity merging threshold of 0.990. After the keyword merging, there were 1574 remaining keywords. The results of the synonymous merging of some keywords are shown in Table 2.

On this basis, we constructed a literature keywords matrix of 1186 × 1574, as shown in Table 3. In the literature keywords matrix, each row represents a literature, each column represents a keyword. The number 1 in the cell means that the literature in the corresponding row contains the keyword in the corresponding column or semantically similar keyword, while the number 0 means that the literature in the corresponding row does not have the keyword in the corresponding column or semantically similar keyword.

#### 3.2.2. Topic Clustering Analysis Using Jaccard Coefficients

**Figure 3:**
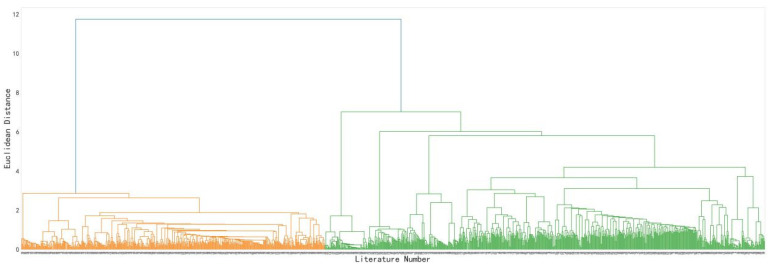
Hierarchical clustering dendrogram.

The Jaccard coefficients between the literatures were calculated based on the literature keywords matrix shown in Table 3, and the literature topics were clustered using the hierarchical clustering method. The hierarchical clustering tree diagram is shown in Figure 3.

When determining the number of topic categories, if there were too few categories, the distribution of the literature was too concentrated. If there were too many categories, the distribution of the literature was too scattered. Both of these are not conducive to exploring research patterns, characteristics, and trends in the field of text sentiment analysis. According to the hierarchical clustering tree diagram, when the clustering distance threshold was 2.85, the current literature in the field of text sentiment analysis could be reasonably classified into 12 topic categories. The final topic clustering results are shown in Table 4.

#### 3.2.3. Key Feature Word Extraction Using Information Gain

In order to further condense the connotation of the topic, it was necessary to extract the key feature words of each type of topic. In this study, we calculated the information gain of 1574 keywords and 12 types of topics, and selected the top five keywords in each topic in terms of information gain as the key feature words. The results are shown in Table 5.

### 3.3. Connotation Analysis of Each Topic

According to the top five high information gain key feature word lists, we have summarized the connotation and research content of each topic category. The 12 categories of topics revealed the current research status of the field in terms of research objects, research methods, research tasks, and research scenarios of text sentiment analysis, respectively. The specific analysis is as follows.

(1) The first topic: Chinese Online Reviews

The key feature word for the first category of topics is “Chinese Online Reviews”. Online reviews are textual data generated by online users sharing their opinions and experiences on social media or consumer platforms, and contain a wealth of feedback on product usage, directly influencing the market performance of the product [20]. Sentiment analysis of online review texts can uncover consumers’ needs for a product from their emotions or attitudes towards product features, which not only benefits merchants’ strategic development, but also helps to support consumers in their purchasing decisions. Therefore, the use of technology to uncover information about consumer needs for product features in online review texts can provide an important basis for market-driven product management decisions [21]. The topic is therefore defined as “Chinese Online Reviews”.

(2) The second topic: Sentiment Analysis Tasks and Scenarios

The key feature word of the second topic category is “Sentiment Analysis Tasks”. This category shows the current application scenarios of text sentiment analysis. Based on the comprehensive analysis of the top five high information gain keywords, it can be seen that text sentiment analysis is a current research hotspot in academia. With the development of computer vision, sentiment analysis based on images has gradually become a potential research hotspot. Due to the richness of the information expressed in images and the diversity of human subjective cognitive factors, the image sentiment analysis task has great development potential [22]. The main sources of the corpus for text sentiment analysis include microblogs, blogs and other social media, and the opinion analysis of online comments is a key application scenario in this field. Therefore, this topic is defined as “Sentiment Analysis Tasks and Scenarios”.

(3) The third topic: Microblog Public Opinion Analysis

The key feature word for the third category is “Microblog”. Microblogging has gradually become a social media platform widely used by scholars in China for text sentiment analysis. Due to its instantaneous, convenient and interesting features, it has attracted a large number of users to obtain information, publish information, express their opinions and express their emotional tendencies by microblogging [23]. Sentiment analysis of microblog content and comments can tap into the attitudes, opinions and demands of the masses on a certain topic and show the opinions of the general public. In addition, the information on personal emotional tendencies embedded in microblog content and comments has the potential to guide public opinion and can influence the psychology of other users, thus driving public opinion. The use of sentiment analysis technology to analyze and identify the emotional attitudes and opinions expressed by netizens at the early stage of the formation of hot topics on microblogs is an effective means for the government to monitor and predict online public opinion, guide the direction of public opinion and control public opinion crises [24]. Therefore, this topic is defined as “Microblog Public Opinion Analysis” research.

(4) The fourth topic: Sentiment Classification

The key feature term for the fourth category of topics is “Sentiment Classification”. Sentiment classification is a sub-task of sentiment analysis, which usually refers to the extraction of subjective evaluations of emotions and attitudes based on the content of an object such as an event, topic or object from textual information, and the use of certain theories and techniques to generalize and summarize the object [25]. The emotion classification task first requires manually annotating text tendencies as a training set, then extracting text emotion features, constructing a classifier through machine learning methods, and finally using the classifier to perform polarity classification on the samples [26]. This is essentially a classification problem. Therefore, various classification models of machine learning are widely used in sentiment classification tasks. This topic is therefore defined as “Sentiment Classification” research.

(5) The fifth topic: Sentiment Lexicon

The key feature word in the fifth category is “Sentiment Lexicon”. The use of emotion dictionaries to determine the polarity of emotions in texts is a traditional research method in sentiment analysis, and many scholars have continued to expand and improve upon them in their research. The construction of sentiment dictionaries is an important basis for text sentiment analysis, but due to the domain characteristics and word habits of different domains, generic sentiment dictionaries are not universally applicable [27]. In addition, due to the increasing volume of data for text sentiment analysis and the rapidly changing online language environment, the traditional sentiment lexicon corpus has shown limitations. Therefore, in recent years, scholars have tended to use the co-occurrence of sentiment words, contextual prior knowledge and text semantic rules and other information to automatically build domain sentiment lexicons [28,29]; so, the topic is defined as “Sentiment Lexicon” research.

(6) The sixth topic: Convolutional Neural Network

The key feature term for the sixth category of topics is “Convolutional Neural Networks”. Convolutional neural networks (CNNs) are one of the classic deep learning network models, originally used in the field of computer vision design. In recent years, scholars have gradually applied them to the field of text sentiment analysis, used to automatically learn hidden features of the text at different granularities, and improved and optimized it, resulting in recurrent CNN [30], extended CNN [31], multi-granularity CNN [32] and so on. These algorithmic models have improved the accuracy of sentiment analysis to a certain extent. However, due to the polysemantic nature of Chinese words, contextual information and the connection features between words, a single neural network is not able to fully extract sentence features and word features [33], so there is still more room for improving sentiment analysis based on improved CNN. Therefore, this topic is defined as “Convolutional Neural Network” research.

(7) The seventh topic: Big Data Mining

The key feature word for the seventh category is “Big Data Mining”. In the era of big data, in the face of the massive amount of information available on the Internet, manual information collection and analysis is no longer sufficient to meet the demand. In addition, it has become an inevitable trend to conduct research in social media and online platforms based on the combination of big data mining and sentiment analysis [34]. With the support of big data technology, mining the opinions, attitudes and emotions of users in social media and online platforms can provide important references for market investment, public management and public opinion guidance. For example, the current research hotspot in the field of financial investment is to capture the sentiment information of investors’ expectations on the development trend of the stock market based on big data to build an investor sentiment index [35]. Additionally, in the field of public administration, sentiment analysis technology based on big data has also become a new way to extract public information to assist in emergency decision-making [36]. Therefore, this topic is defined as “Big Data Mining” research.

(8) The eighth topic: Deep Learning

The key feature word for the eighth category is “Deep Learning”. Deep learning is rapidly developing and becoming one of the most popular research methods in the field of natural language processing. Its research and application in the field of sentiment analysis are becoming more and more widespread, and it has become an indispensable part of the field, greatly improving the accuracy of sentiment analysis. The authors have used deep learning models such as recurrent neural networks (RNN), convolutional neural networks (CNN), capsule networks, long short-term memory networks (LSTM), gated recurrent units (GRU), bi-directional long short-term memory networks (Bi-LSTM), pre-trained language models, combined with attention, positional encoding, and other deep learning models. A variety of excellent sentiment analysis models have been built to optimize feature extraction and sentiment classification in sentiment analysis [37]. This topic is therefore defined as “Deep Learning” research.

(9) The ninth topic: Aspect-Based Sentiment Analysis

The key feature term for the ninth topic category is “Aspect-Based Sentiment Analysis”. Aspect-based sentiment analysis is also known as fine-grained sentiment analysis or attribute-based sentiment analysis.

Compared to traditional coarse-grained sentiment analysis, aspect-level sentiment analysis can provide more comprehensive, in-depth, and fine-grained analysis, which is a significant benefit in sentiment analysis research. Aspect-based sentiment analysis consists of various basic subtasks, such as aspect term extraction (ATE), which extracts target entities and phrases in sentences, and opinion term extraction (OTE), which extracts words with subjective opinions. In recent years, research has focused on solving these tasks individually or combining two tasks, such as aspect term polarity common extraction (APCE), aspect opinion common extraction (AOCE) and aspect opinion pair extraction (AOPE). The topic is therefore defined as “Aspect-Based Sentiment Analysis” research.

(10) The tenth topic: Feature Extraction

The key feature word for the tenth category of topics is “Feature Extraction”. For Chinese text sentiment analysis, feature extraction and sentiment feature learning are required to determine the sentiment polarity of the text. Feature extraction is a subtask of sentiment analysis that is mainly used to extract opinion targets from research text, and is divided into explicit feature extraction and implicit feature extraction. Due to the influence of factors such as the absence of key emotional features, expression carrier forms, and cultural customs, Chinese implicit sentiment classification tasks are more difficult than those in other languages [38], and implicit feature extraction in Chinese has become a research focus and difficulty for domestic scholars. Therefore, this topic is defined as “Feature Extraction” research.

(11) The eleventh topic: Multimodal Sentiment Analysis

The key feature word for the eleventh category of topics is “Multimodal”. With the change in mainstream media, short videos, mainly represented by Douyin and Kuaishou, have become an important medium for the public to express their opinions. Videos include modalities such as sound and images, which are typical multimodal data and provide more information than single-modal data [39]. Applying multimodal learning to sentiment analysis has become a core research topic in the field of natural language processing [40]. Unlike single-modal learning for sentiment analysis, multimodal learning can integrate related semantic information from different modalities, usually by combining information from two or more modalities to identify sentiment. This is beneficial for achieving fine-grained sentiment mining, identifying ironic emotions in user comments, and achieving a more accurate sentiment analysis [41]. Currently, the technology for multimodal sentiment analysis in China is still in the early stage of development, and there is still considerable potential for development in theoretical methods and practical applications. Therefore, this topic is defined as “Multimodal Sentiment Analysis” research.

(12) The twelfth topic: Word Embedding

The key feature word for the twelfth category of topics is “Word Embedding”. Word embedding can mine the hidden semantic relationships between words from large-scale corpora through contextual information [42], and has been widely applied in feature extraction and sentiment classification. In natural language processing tasks, the representation of words in computers is the first consideration. Therefore, word vector technology is fundamental work in the field of natural language processing. Currently, neural-network-based word vector calculation technology, such as Word2Vec or GloVe, is relatively mature. In the field of sentiment analysis, scholars focus on improving and optimizing traditional word vector technology, such as the feature-weighted word vector proposed by Gao [43], which uses word vector models to accurately characterize the sentiment information of words and plays an important role in improving sentiment classification performance. Cao et al. proposed a multi-word vector fusion sentiment classification framework based on mutual learning, which can fully utilize the information in ordinary word embedding, domain-specific word embedding, and sentiment word embedding to improve classification performance [44]. Therefore, this topic is defined as “Word Embedding” research.

### 3.4. Annual Distribution of Literature and Research Trend Analysis

#### 3.4.1. Annual Distribution of Literature in Each Topic

In order to more accurately grasp the research hotness and future trends of each topic in the field of sentiment analysis, we constructed a table of the literature distribution and average annual growth rate of each topic in the field of sentiment analysis from 2012 to 2022 was constructed, as shown in Table 6.

According to Table 6, the overall trend in the volume of literature on various topics in the field of sentiment analysis can be divided into two stages. The first stage is from 2012 to 2016, when the literature related to sentiment analysis was small and growing slowly, and was in the initial development period. The second stage is from 2017 to 2022, when sentiment analysis research gradually entered the exploration and development period and rapidly heated up, with a linear growth of research results.

On the basis of the two time periods, the subject distribution matrices for the two time periods were constructed based on the two dimensions of subject literature volume and average annual growth rate of the literature with reference to the Boston Consulting Group Matrix (BCG Matrix) idea. The two time intervals used in calculating the cumulative literature volume and the average annual growth rate of literature for each theme were 2012 to 2016 and 2017 to 2022, respectively. In each stage, the threshold value for the volume of literature was set as the volume of literature in a balanced state of development for each topic, i.e., the total volume of literature divided by the number of topics in that stage, and the threshold value for the volume of literature was calculated. In addition, the threshold value for the average annual growth rate of literature was set as the average annual growth rate of the total literature of each topic in that stage. The calculation results are shown in Table 7.

#### 3.4.2. Topic Distribution Matrix and Research Trend Analysis

**Figure 4:**
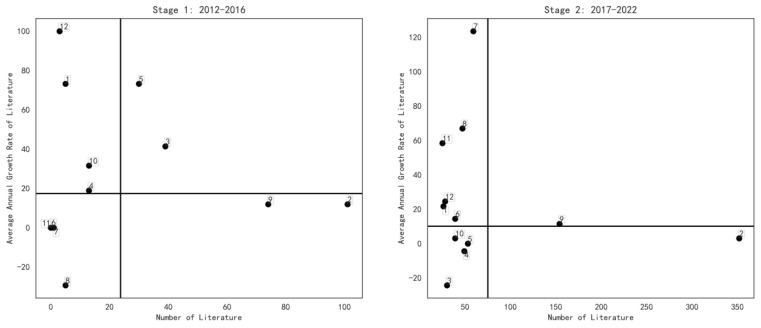
Two-stage-based sentiment analysis topic distribution matrix.

Based on Table 7, the four quadrants of the sentiment analysis study were constructed by dividing the literature volume into two areas of high and low volume, and the average annual growth rate into two areas of high and low volume, based on the critical values of the volume and average annual growth rate. The first quadrant is a combination of (High Volume, High Growth), in which topics in this quadrant have a high volume and growth rate, indicating that the topic is receiving extensive and sustained attention from scholars at this stage. The second quadrant is (Low Volume, High Growth), where topics in this quadrant have a low cumulative volume of publications but show high growth and are the focus of current or future research. The third quadrant (Low Volume, Low Growth) is a combination of low volume and no significant growth, indicating that the topic has not been studied in depth and is not receiving much attention. The fourth quadrant (High Volume, Low Growth) is a combination of high volume and low growth rates, with a large number of cumulative research results, but low average annual growth rates. Based on the above four quadrants, a sentiment analysis topic distribution matrix was constructed by combining the cumulative volume of the literature and the average annual growth rate of the literature for each topic, as shown in Figure 4.

According to Figure 4, comparing the research trends of various topics in the field of sentiment analysis in two phases, we can see that the average annual growth rate of the literature in the first category “Chinese Online Review” and the 12th category “Word Embedding” decreased in the second phase, but the volume of literature increased still increased in the second phase. This indicates that the research on these two types of topics in the field of emotional analysis is still highly popular. The average annual growth rate of the literature for category 2 “Sentiment Analysis Tasks and Scenarios” declined in the second stage, and the growth of the literature slowed down, but it is still in the fourth quadrant, which indicates that the cumulative research results of this category are large and the research trend is very stable. Category 3 “Microblog Public Opinion Analysis”, category 4 “Sentiment Classification”, category 5 “Sentiment Lexicon” and category 10 “Feature Extraction” moved from outside the third quadrant in the first stage to the third quadrant in the second stage. Although the volume of literature has a stable or rising trend, the average annual growth rate of the literature has decreased, indicating that the research enthusiasm for these four categories of topics has fluctuated, showing a slight decline. Compared to the first stage, category 6 “Convolutional Neural Network”, category 7 “Big Data Mining”, category 8 “Deep Learning” and category 11 “Multimodal Sentiment Analysis” are all in the second quadrant. The volume of literature and the average annual growth rate of the literature are obviously on the rise, indicating that these four topics have gradually attracted the attention of researchers and become the focus of current or future research, taking into account the development of social reality, current technological conditions and other factors. Category 9, “Aspect-Based Sentiment Analysis”, shifted from quadrant 4 in phase 1 to quadrant 1 in stage 2, with a significant doubling of the literature, suggesting that this category has been a topic of great research interest in the field of sentiment analysis and has never faded from researchers’ attention.

## 4. Conclusions

Over the past decade, there has been significant progress in the field of Chinese text sentiment analysis, with a tremendous increase in the number of published papers.

Different from the traditional bibliometric methods, our study used the FastText model to calculate the keyword embedding, merged synonymous keywords, and combined the Jaccard coefficient for hierarchical clustering to form 12 types of research topics. Then, we used information gain to extract topic feature words, and, finally, combined with literature time series analysis, proposed a two-stage sentiment analysis research four-quadrant matrix. Based on this comprehensive research system, this study summarized the research fields, methods, and objects of current text sentiment analysis, revealed the research frontiers and hotspots of sentiment analysis and predicted future research trends. The specific conclusions were as follows:

First, in terms of the research field of sentiment analysis, a significant amount of research has been conducted on public opinion analysis based on social media online comment data, and future research should expand sentiment analysis research in specific scenarios. The current research data on sentiment analysis mainly comes from online comments on social media such as Weibo, Douban, and Zhihu. Future sentiment analysis research can be expanded to other professional fields, such as stock market forum data-based evaluations of investor sentiment, tourist satisfaction evaluations based on travel website comments, and online entertainment product consumption behavior research based on video bullet screen data. Wider research can enable sentiment analysis to play its profound application value in finance, politics, business, service industries, and healthcare.

Second, from the perspective of the research level in sentiment analysis, aspect-based sentiment analysis is currently the research focus and has broad prospects. In actual application scenarios, users not only need to identify the opinions contained in a document or a sentence, but also need to identify the objects expressed or evaluated by the opinions or sentiments, as well as the specific opinion tendencies expressed for these objects. In this case, scholars should gradually delve into the field of fine-grained sentiment analysis, researching aspect-based, attribute-based, topic-based, and entity-based sentiment extraction and classification. Although the research on aspect-level sentiment analysis has been highly popular and has shown good overall development trends in recent years, the long-standing difficulties between scientific research and practical application still exist, mainly including problems such as irony recognition, anaphora and coreference resolution, and semantic disambiguation. Therefore, these are key areas of concern and urgent problems to be addressed in future sentiment analysis research.

Third, from the perspective of research methods in sentiment analysis, the application effectiveness of sentiment lexicons, traditional machine learning, and deep learning in sentiment analysis should be improved. With the rapid rise of deep learning, breakthroughs were made in many areas such as image, speech, and text. The use of deep learning to solve problems related to sentiment analysis has become the current mainstream research method and has played a significant role in promoting sentiment analysis technology. Although the emerging deep learning methods have, to some extent, made up for the deficiencies of traditional methods, their algorithm implementation difficulty is high, and they have stringent requirements for training datasets. Therefore, future research on sentiment analysis methods should focus on the integration of sentiment lexicons, traditional machine learning, and deep learning methods, and further optimize the existing deep learning models to improve the accuracy of sentiment analysis.

Fourth, from the perspective of research objects in sentiment analysis, multimodal fusion sentiment analysis and cross-modal sentiment analysis should be promoted. Currently, sentiment analysis is mostly based on online textual comment data, but with the rise of audio and video media, multimodal data with significant research value have emerged. Currently, it is still challenging to extract features from each modality effectively, and it is also challenging to fuse these features effectively. In the future, multimodal sentiment analysis and cross-modal sentiment analysis based on image, speech, video, and other data should be strengthened.

There are some limitations to this study. In the future, the use of dynamic word vector models will be explored. Compared with the static word vector model of FastText, the dynamic word vector model, such as a pre-trained deep learning model, can make fuller use of contextual information. Therefore, it can characterize the meaning and semantic relationships of keywords more accurately, leading to the higher accuracy of the keyword merging process. Furthermore, deep neural network learning algorithms, such as the sentence vector model, will be explored to learn topic features from text and add topic information with global features to word vectors, which can improve the concentration of topic identification and clustering.

## Figures and Tables

**Table 1:** Semantic similarity matrix of keywords.

Keywords	ALBERT	Attention Mechanism	......	CRF Model	Attention-Highway
ALBERT	1.000	0.991	......	0.954	0.884
Attention Mechanism	0.991	1.000	......	0.952	0.940
......	......	......	......	......	......
CRF Model	0.954	0.952	......	1.000	0.871
Attention-Highway	0.884	0.940	......	0.871	1.000

**Table 2:** Examples of synonymous subsumption of keywords.

Keywords	Similar Keywords and Similarities
Word Vector	Word Granularity Word Vector (0.997), Char-Word Representation (0.995), Word Vector Filling (0.996)
Context	Global Context (0.992), Context Enhancement (0.990), Context-Aware (0.990), Context Entropy (0.996), Contextual Constraints (0.993)
Aspect-Level Sentiment Analysis	Aspect-Based Sentiment Analysis (0.991), Multi-Aspect Sentiment Analysis (0.992), Attribute Sentiment Analysis (0.992), Attribute-Level Sentiment Analysis (0.997), Aspect-Level Sentiment Analysis (0.997)
Topic Probability Model	LDA Model (0.995), Theme-Emotion Mining Model (0.999), Topic Sentiment Model (0.995), Topic Sentiment Mixture (0.993), Time-Aware Sentiment-Topic Model (0.996), Probability Topic Model (0.993), Topic Model (0.996)
Attention Mechanism	Interaction Attention Mechanism (0.992), Positional Attention Mechanisms (0.995), Bidirectional Attention Mechanism (0.995), Dual-Way Attention Mechanism (0.998), Dual Attention Mechanism (0.996), Sentry Attention Mechanism (0.998), Multi-Head Attention (0.991), Multi-Attention Mechanism (0.998), Locality Sensitive Hashing Attention (0.993), Sparse Self-Attention Mechanism (0.993), Self-Attention Mechanism (0.997), Self-Attention (0.991)

**Table 3:** Literature keywords matrix.

Literature Number	3D Convolutional Neural Networks	BILSTM	…	Thematic Sentiment Analysis	Interaction Attention Mechanism	Aspect-Level Sentiment Analysis
1	0	0	…	0	1	0
2	0	1	…	0	0	0
3	0	0	…	1	1	1
…	…	…	…	…	…	…
1186	0	0	…	0	0	0

**Table 4:** Literature clustering results.

Category Number	Number of Literature	Percentage (%)	Category Number	Number of Literature	Percentage (%)
1	31	2.61	7	60	5.06
2	453	38.20	8	52	4.38
3	69	5.82	9	228	19.22
4	62	5.23	10	52	4.38
5	83	7.00	11	25	2.11
6	40	3.37	12	31	2.61

**Table 5:** Top five high information gain keywords.

Category Number	Keywords and Information Gain (×10^−2^)	Category Topic
1	Chinese Online Reviews (4.194), Multi-Attribute Online Review Decision Making (4.153), Online Review of Cigarette (4.115), Online Reviews Mining (4.077), Online Review Text (3.973)	Chinese Online Reviews
2	Sentiment Analysis Task (14.634), Analysis of Public Sentiment and Emotion (14.500), Image Sentiment Analysis (13.104), Thematic Sentiment Analysis (12.903), Text Sentiment Analysis (12.432)	Sentiment Analysis Tasks and Scenarios
3	Microblog (9.305), Emoticon (9.305), Microblog Data (9.181), Public Opinion on Microblog (9.181), Weibo Rumors (9.181)	Microblog Public Opinion Analysis
4	Sentiment Classification (6.375), Sentiment Classification Annotation (6.308), Sentiment Classification Model (6.276), Image Sentiment Classification (6.212), Text Sentiment Classification (6.151)	Sentiment Classification
5	Sentiment Lexicon (9.547), Financial Sentiment Lexicon (9.478), Sentiment Lexicon Construction (9.410), Highly Credible Lexicon (9.344), Semantic Lexicon Matching (9.280)	Sentiment Lexicon
6	3D Convolutional Neural Networks (2.617), Convolutional Neural Networks Model (2.123), Bidirectional Sliced Gated Recurrent Unit (0.547), Bidirectional Gated Recurrent Neural Network (0.520), Deep Neural Network (0.485)	Convolutional Neural Network
7	Big Data Mining (1.988), Analysis of Public Sentiment and Emotion (1.795), Sentiment Analysis (1.782), Chinese Microblog Sentiment Analysis (1.767), Text Sentiment Analysis (1.414)	Big Data Mining
8	Deep Learning (2.252), Deep Learning Hybrid Model (2.190), Natural Language Processing (1.063), Natural Language Processing Technology (1.051), End-to-End (0.230)	Deep Learning
9	Aspect-Level Sentiment Analysis (7.247), Thematic Sentiment Analysis (6.462), Sentiment Analysis Task (6.388), Image Sentiment Analysis (5.970), Fine-Gained Sentiment Analysis (5.231)	Aspect-Based Sentiment Analysis
10	Feature Extractor (1.939), Local Feature Extraction (1.889), Text Feature Extraction (1.889), Customer Features Extraction (1.889), Sentiment Feature Extraction (1.651)	Feature Extraction
11	Multimodal Learning (4.439), Multimodal Application (4.090), Multimodal Fusion Architecture (4.090), Multimodal Network (3.990), Multimodal Data (3.886)	Multimodal Sentiment Analysis
12	Word Embedding Model (4.373), GloVe Word Vector (4.325), Sentiment Enhanced Word Vector (4.279), Feature Weighted Word Vector (4.115), Location-Weighted Word Vector (4.006)	Word Embedding

**Table 6:** Annual distribution of research topics.

Category Number	Category Topic	2012	2013	2014	2015	2016	2017	2018	2019	2020	2021	2022	Total	Average Annual Growth Rate (%)
1	Chinese Online Reviews	0	0	1	1	3	3	3	4	4	4	8	31	29.68
2	Sentiment Analysis Tasks and Scenarios	14	15	21	29	22	48	46	64	73	65	56	453	14.87
3	Microblog Public Opinion Analysis	2	6	12	11	8	8	6	7	3	4	2	69	0.00
4	Sentiment Classification	2	1	4	2	4	5	9	7	13	11	4	62	7.18
5	Sentiment Lexicon	1	5	10	5	9	7	12	11	6	10	7	83	21.48
6	Convolutional Neural Network	0	0	0	0	1	0	0	6	13	11	9	40	44.22
7	Big Data Mining	0	1	0	0	0	0	1	3	9	21	25	60	43.00
8	Deep Learning	0	0	2	2	1	1	3	9	11	10	13	52	26.36
9	Aspect-Based Sentiment Analysis	14	9	11	18	22	18	27	24	30	24	31	228	8.27
10	Feature Extraction	2	0	2	3	6	6	7	4	8	7	7	52	13.35
11	Multimodal Sentiment Analysis	0	0	0	0	0	1	0	2	3	9	10	25	58.49
12	Word Embedding	0	0	0	1	2	1	6	8	6	4	3	31	16.99
Total		35	37	63	72	78	98	120	149	179	180	175	1186	14.35

**Table 7:** Literature volume thresholds and growth rate thresholds for both stages.

Time Interval	Number of Literature	Literature Volume Thresholds	Growth Rate Thresholds
stage 1: 2012~2016	285	23.75	17.38%
stage 2: 2017~2022	901	75.08	10.15%

## Data Availability

All data that support the findings of this study are available from the corresponding author upon reasonable request.
